# Correction of Potassium Fertigation Rate of Apple Tree (*Malus domestica* Borkh.) in Central Russia during the Growing Season

**DOI:** 10.3390/plants9101366

**Published:** 2020-10-15

**Authors:** Andrei I. Kuzin, Natalia Ya. Kashirskaya, Anna M. Kochkina, Alexey V. Kushner

**Affiliations:** 1 I.V. Michurin Federal Scientific Centre, 393774 Michurinsk, Russia; kashirskaya@fnc-mich.ru (N.Y.K.); ms.anna.step@mail.ru (A.M.K.); alexkoushner@mail.ru (A.V.K.); 2 Department of Horticulture, Michurinsk State Agrarian University, 393760 Michurinsk, Russia

**Keywords:** potassium leaf and fruit status, calcium leaf and fruit status, yield, soil potassium and calcium, K/Ca ratio

## Abstract

The proper use of potassium fertilizer can stimulate a significant yield increase. However, the application of excessively high rates of potassium can reduce the availability of soil calcium for apple trees. The potassium fertigation rate must meet the apple tree’s requirements so that the applied fertilizers can be absorbed by the roots as much as possible. Crop load in apple orchards sometimes varies significantly in different years. The potassium content in apple fruits is relatively high, and the maximum requirement for this nutrient occurs when fruits grow and ripen. Different crop loads at that time mean the various demands of trees and need for changing application rates for this nutrient. The investigation was carried out in the experimental orchard of I.V. Michurin Federal Scientific Centre (Michurinsk, Russia) in 2016 and 2017 (52.885131, 40.465613). We studied seasonal changes of potassium and calcium contents in soil, fruits, and leaves and their relationship with yield during the research. We paid much attention to the potassium rate shift on its content in leaves and fruits and cultivars “Lobo” and “Zhigulevskoye” yield. If the potassium application rate changes according to the actual crop load, it stimulates the yield growth or (if the crop load was relatively low) the reduction of the rate did not lower the productivity. Moreover, we studied the relationship between potassium and calcium nutrition. The decrease in potassium fertigation rate increased the availability of soil calcium. It was the reason for fruit calcium concentration enlargement and mitigation of the K/Ca ratio. We also specified some parameters for soil–leaf diagnosis for potassium nutrition during the growing season.

## 1. Introduction

Potassium plays a crucial role in plants. It is believed that the apple tree needs potassium even more than nitrogen [1]. Potassium has a higher specificity compared to other nutrients. It has an essential role in the growth, development, and fruiting of apple trees, but it is not part of the molecules of organic substances in plants. It practically does not form compounds with covalent bonds in the body. At the same time, it participates in almost all physiological and biochemical processes: photosynthesis, breathing, transpiration, turgor, metabolism, transport of substances in plants, etc. [2,3]. The high content of some macronutrients in the soil can change the availability of others. Thus, an increase in potassium rate can lead to a specific decrease in nitrogen in plant leaves. Holb et al. [4] observed such phenomena in the field when leaf nitrogen status decreased with increasing potassium rate. In sand culture, the nitrogen concentration in leaves of 6-year-old plants Gala/M26 decreased because of high potassium rates [5]. Similar results were also obtained by other authors on different apple cultivars: with eight cultivars in the “Red Delicious” and eight cultivars in the “Virginia” groups, cv. “Gala”, “Golden Delicious”, and “Fuji” [6,7]. At the same time, a boost of nitrogen supply can stimulate the decrease of potassium leaf status. Thus, growing orange plants in a sandy culture increased nitrogen nutrition and reduced the potassium content in the leaves on low potassium level, but with a high potassium supply, there was no such effect [8].

Leaf phosphorus concentration did not depend on different levels of soil potassium during the growing season [4,9]. Seasonal variations of phosphorus leaf status depend more on the scion and rootstock [10]. In these authors’ study, an increase in potassium nutrition level led to a decrease in leaf phosphorus content of the A2 rootstock in the middle of August. Simultaneously, an increase in the phosphorus leaf status of some other tree species was noted with the rise of soil potassium [11].

The problem of the relationship between potassium and calcium nutrition in this series is separate. The maximum demand for phosphorus and nitrogen, as a rule, does not coincide with the maximum absorption of potassium, while calcium and potassium overlap in time. It is one of the possible reasons that in the literature there is conflicting information about the rates of potash fertilizers and their effect on the yield and quality of apple fruits [12]. In this regard, some questions arise about potassium nutrition: What is the optimal level of potassium content in apple leaves and the best sampling timing? In a nursery, without crop load, the potassium leaf status is relatively stable, and, as a rule, its content in the leaves matches the soil concentration of this nutrient [13]. Mature trees have potassium reserves in the wood [14]. The significant effect on potassium leaf status makes crop load when the concentration of this nutrient in leaves significantly decreased because of fruit growth and ripening, but after harvest, the potassium leaf status recovering [5,13]. There is some evidence that the leaf potassium level did not increase after harvest [4], but it could be explained by low soil available potassium.

The main need for potassium absorption begins during the growth and development of the fruit [2]. Leaf potassium decreases due to its high mobility and transporting to fruits in this period [13]. In the existing recommendations for Central Russia, the potassium level in apple leaves should be 1.3–1.5% d.m. when sampling late July-middle August (100–120 days after bud break [15]). Chang [2] cited data of Stiles and Ride—1.35–1.85% (60–70 days after petal fall). After the intensive shoot growth stage, when trees require nitrogen, the absorption by apple plant potassium and calcium is increased, which is extremely important for the subsequent storage of fruits.

Unlike potassium, calcium has low mobility in plants and is stored with the growing season in leaf vacuoles as oxalate [16]. In the investigation of Cheng and Raba [5], the content of calcium in the leaves immediately after blossoming slightly decreased and increased until the end of the vegetation period. The seasonal changes of calcium concentration in leaves of M26 rootstock at mother plantation were characterized by increased leaf calcium by the end of the vegetation period [17]. The content of exchangeable calcium (like potassium) is relatively high in chernozem soil. However, calcium absorption is often depressed because of relatively high concentrations of potassium [18]. The growing season in the Tambov region usually begins near the end of April, and late-season apple cultivars are harvested at the end of September–beginning of November. Because of this relatively short fruit maturation period, the overlap of calcium and potassium demand will be more substantial. The application of high potassium rates can reduce the amount of absorbed calcium and its concentration in fruits. Limiting the absorption of calcium by plant roots leads to a “strong competition” for this nutrient between shoots and leaves on the one hand and fruits on the other [19,20].

The content of various nutrients in plant organs more or less depends on the supply of potassium. In this series, calcium stands out because it provides resistance to physiological disorders during storage. Therefore, the soil potassium fertilization must not have a strong inhibitory effect on the plant roots’ uptake of calcium. Our study aimed to investigate the possibility to correct potassium fertigation rate during the growing season according to early sampled leaf potassium status and actual crop load to avoid excessive fertilization and reducing the availability of soil calcium.

## 2. Materials and Methods

### 2.1. Research Location and Methods

The study was carried out in the experimental orchard of the I.V. Michurin Federal Scientific Centre in 2016 and 2017. The experiment was done in the orchards (52.885131, 40.465613) with drip irrigation (2222 tr. ha^−1^, cultivars “Lobo” and “Zhigulevskoye” grafted on the rootstock B396). Trees of cv. “Zhigulevskoye” were planted in 2007, cv. Lobo, in 2010. The experiment was arranged in randomized blocks with 5 trees and 4 replicates. Agrochemical properties of trial plot soil of cv. “Zhigulevskoye”: leached meadow-chernozem, humus 2.9%, the amounts of absorbed bases—26.8 meg 100 g^−1^ soil, soil hydrolytic acidity (10–40 cm)—5.3 meg 100 g^−1^ soil, CEC—32.1 meg 100 g^−1^ soil The reaction of topsoil was slightly acidic pH_KCl_ = 5.4. The content of easily hydrolyzed nitrogen—152.4; mobile phosphorus—125.3; exchangeable potassium—142.4 mg kg^−1^ soil. Agrochemical properties of trial plot soil of cv. “Lobo”—leached meadow-chernozem, humus 2.4%, the amounts of absorbed bases—24.6 meg 100 g^−1^ soil, soil hydrolytic acidity (10–40 cm)—5.8 meg 100 g^−1^ soil, CEC 30.4 meg 100 g^−1^ soil. The reaction of topsoil was slightly acidic pH_KCl_ = 5.1. The content of easily hydrolyzed nitrogen—128.7; mobile phosphorus—115.9; exchangeable potassium—139.2 mg kg^−1^ soil. The soil analyzes were done before the experiment. The aggregates that are larger than 0.25 mm are so cold “agronomically valuable units”, less than 0.25 mm is sand, dust, and physical clay. The soil aggregation of cv. “Zhigulevskoye” plot was better in amount of valuable aggregated (Table 1).

The application rate for fertigation in research treatment was calculated based on the content of primary nutrients in the soil of experimental plots and taking into account the optimal value for the Tambov region, defined by us earlier: N_25_P_20_K_30_ (data are in kg ha^−1^ active ingredients) [21]. We divided the treatments in our research into two groups: G1 and G2. According to the calculated fertigation rate, we applied fertilizers during the growing season without any changes in G1. In the variant group G2, using the same level of nitrogen and phosphorus, we changed the potassium rate after determining the crop load. We discovered the crop load by visual counting clusters, and then we calculated small fruits after fruitlet drop (fruit size: 25–30 mm) and fruits (40–45 mm). Then, we corrected the potassium fertigation rate based on actual crop load. As the Control in our experiment for both groups of variants, we used the treatment without the application of potassium: N_20_P_15_ (Table 2).

G1 trial plots were the same in both years of the research. For G2 experimental sites, the potassium fertigation rate was different in 2016 and 2017 because, during the growing season, we corrected the calculated rate according to actual crop load. For fertigation, we used ammonium nitrate, ammonium phosphate, potassium monophosphate, potassium sulfate, as well as products (with formula NPK+Mg) 13.40.13, master 18.18.18 + 3, master 3.11.38 + 4 with the complex of microelements depending on the growing stage. We also made foliar nutrition sprayings in all experimental treatments, including Control (Table 3).

We did 7 foliar treatments with calcium-containing products (17% Ca, 10% total N, 0.8% Mg, 0.02 Zn, 0.02% Cu, 0.05% B, 0.001% Mo) on “Zhigulevskoye” trees, and 9 on “Lobo” plants as this later ripening cultivar. The sprayings were begun when fruits were 20–25 mm and finished in 2 weeks before harvesting.

Leaves and soil were sampled monthly from May 30 till September 30. We determined in leaves total potassium and calcium content on flame photometer (FPA-2.01 (Russia). Soil samples were analyzed on the content of exchangeable potassium (flame photometer FPA-2.01) and exchangeable calcium by complexometric method (titration of calcium with Trilon B at pH 12.5–13.0 using murexide as an indicator) [22]. The data obtained in the experiment were analyzed statistically by Fisher’s method. We calculated the Lowest Significant Difference (LSD) between various treatments (*p* < 0.05). The differences are higher than the computed LSD value are considered significant. The correlation coefficient was calculated according to the Person’s method.

### 2.2. Weather Conditions in 2016 and 2017

The air temperature was slightly higher than usual in April 2016 (Table 4), that was the reason for the earlier beginning of growing season by both cultivars 12 April. The first half of the vegetation period was complicated by significantly large precipitation than the average for 50 years. Primarily, it was characteristic of April and May, which undoubtedly affected plants’ development during these months (Table 5). Air temperatures significantly exceed the average in July and August, but the amount of precipitation was considerably lower than usual in these months. Lack of moisture in the summer months is typical for our region, but in an orchard with drip irrigation, this could not have a strong negative impact on the state of trees. Cv. “Zhigulevskoye” fruits were harvested on 23 August and cv. “Lobo” 15 September in 2016.

In 2017, the air temperature conditions at the beginning of the growing season did not differ from the average annual rate except cooler May. There were no frosts, although there were nights with low temperatures of 1.5–4.5 °C. Such a situation was directly after blossoming, and this could increase the drop of fruitlets. The amount of precipitation in the growing season 2017 was more than usual, but its distribution through the vegetation period differs as it was in 2016. It was a lack of rainfall in May, June, and July, whereas their amount was more than the usual in August. In the drip-irrigated orchard, the lack of precipitation is not a big problem. But the periods of waterlogging, especially on heavy loamy chernozem (which has a high water-holding capacity), could make a substantial negative impact on roots. The growing season 2017 began later than in the year before 2 April, cv. “Lobo” and 23 April cv. “Zhigulevskoye”. The harvest time for cv. Zhigulevskoye was 28 August and for cv. “Lobo” was 21 September.

Despite some negative periods associated with waterlogging, which were not long, weather conditions during the research favored the growth and fruiting of apple trees.

## 3. Results

### 3.1. Development of Some Productivity Components

Cv. “Lobo” and “Zhigulevskoye” do not differ in the propensity to alternative bearing, especially at a relatively young age. The blossoming was comparatively abundant in warm weather in 2016, but then because of waterlogging, was a substantial drop of fruitlets (Table 6). Initially, the potassium application rate in both groups was the same. According to this rate, we fertigated G2 variants before counting small fruits (20–25 mm). The application rate in G2 was reduced according to the number of dropped fruitlets. Then, we cut the potassium rate once again after counting the number of fruits (40–45 mm) and the leaf potassium status. In the tables, we show the final season fertigation rate for G2.

The number of clusters on “Lobo” trees was a little bit lower than cv. “Zhigulevskoye”, but we decided not to apply different rates on various cultivars to avoid complicating of the experiment design. Nevertheless, note that by developing a complete algorithm for changing the potassium rate based on crop load such a difference can be taken into account.

We repeated our action algorithm from the previous year in 2017, when the blossoming was much more intensive (Table 7). Nevertheless, we decided not to change the potassium application rate in G1 for the reasons mentioned above. However, according to the increase in the number of clusters, small fruits, and fruits, we enlarged the potash fertigation in G2.

The number of clusters of both cultivars didn’t vary significantly in 2017 in all treatments. The increase of the potassium application rate stimulated the growth of the fruit number (40–45 mm). In “Zhigulevskoye” orchard, we saw a significant difference only when applied K45 (G1) and K50 (G2). “Lobo” trees have a slightly better response on potash fertigation. The fruit number significantly increased in K35 and K40 (G1), and K40 and K50 (G2) treatments compared to the Control and variants with lower rates.

### 3.2. Impact of Potassium Nutrition on the Amount of Yield

The yield varied significantly in different years of the study (Table 8). Such variations could be explained because of the alternative bearing problem, and the unfavorable weather conditions in 2015 when flower buds were differentiated. In 2016, the significant yield increase of cv. “Zhigulevskoye” occurred only in the K45 treatment (G1). In G2 with reduced potassium rates, the considerable yield increase compared to the Control occurred in the K25 and K30 treatments. Therefore, reducing the potassium fertilizer amount according to the actual crop load didn’t negatively impact. The yield of cv. “Lobo” trees was lower than “Zhigulevskoye” because this orchard was three years younger. However, on the “Lobo” plantation, potassium fertilizer’s application stimulated a significant yield increase in all treatments. We want to emphasize that the stable yield 25–35 ha^−1^ for our region is quite a good result. In G1 (where potash fertilizers were made without taking into account actual crop load), the highest yield was in the treatment K25. Still, there were no statistically confirmed differences between the variants with potassium fertigation. Cv. “Lobo” in G2 had the lowest yield corresponded to the minimum potassium rate, which was calculated according to the actual crop load. The maximum output was in K30 treatment, significantly higher than in all other variants in this year.

Potassium rate K45 provided a significant yield increase of cv. “Zhigulevskoye” compared to the Control and other G1 variants in 2017. In G2, the yield was significantly more than in the Control, and there was no difference between K30 and K40. The application rate of potassium 50 kg ha^−1^ provided the largest yield of cv. “Zhigulevskoye” in 2017. Potassium fertigation in all treatments with cv. “Lobo” made a significant impact on productivity compared to the Control in 2017. In G1, the maximum productivity was in K45, but the difference with K35 was not essential. In G2, the increase of potassium rate stimulated the yield rise corresponded to the amount of fertilizer. It was no significant difference in yield between the K30 and K40. The maximum output, significantly higher than in all other treatments on cv. “Lobo” was in the K50 variant.

### 3.3. Seasonal Changes of Exchangeable Potassium and Calcium in Soil of the Experimental Plots

Seasonal soil exchangeable potassium (Figure 1 and Figure 2) and calcium (Table 8) changes are presented only for G1 variants because they took the same experimental plots in both years. The changes in soil potassium concentration were similar in different plots. The content of potassium increased up to the end of June in all treatments, including the Control. In this period, the plant need for potassium was not maximal and average day air (and soil respectively) temperature increased from 13.8 °C to 22.0 °C; soil humidity was quite favorable, so more potassium ions were free from soil particles. July was drier, but at this time, plant requirements in potassium significantly increased. Because of this, the concentration of soil exchangeable potassium reduced. The most substantial decrease in potassium amount we saw in August when fruits matured. After “Zhigulevskoye” fruits were harvested on 25–26 August 2016, the potassium content recovered to the level at the beginning of vegetation.

Seasonal changes in the soil exchangeable potassium content had an utterly different character in 2017. The nutrient concentration changed in treatments with high application rates approximately like in the previous year. There was no potassium content increase in June in variants with the lower rate and in the Control. In these treatments (K0 and K25), were a significant decrease in the soil potassium amount in July 2017, then it slightly increased in August and did not have any essential changes in September.

Dynamic changes in the soil exchangeable potassium content in cv. “Lobo” plots in 2016 were generally similar to the variations of this nutrient concentration on the cv. “Zhigulevskoye” sites (Figure 2). However, there were also significant differences: the content of the nutrient was lower; application of the lowest potassium rate did not make a significant impact on the concentration value compared to the Control (K0); only the application of high rates makes the essential increase in the soil potassium. Timing of the fruit ripening also impacted dynamic changes “Lobo” fruits were harvested a month later than “Zhigulevskoye”. The soil potassium content on 30 September in “Lobo” site was generally the same as August’s end in “Zhigulevskoye” plot.

In 2017, the variations of soil potassium concentration among the “Lobo” plots were more significant. The lowest potassium application rate didn’t have a noticeable effect on its soil concentration, like in 2016. Nutrient content changed by applying the K35 rate as in 2016 but did not recover after harvesting. The dynamic changes in the soil potassium using the maximum rate (K45) during the growing season were erratic. The lowest potassium content was in this year in June, but usually, at that time was the largest concentration of the season. The concentration of soil potassium then increased to the end of July, which also was unexpected.

The soil calcium fertilizer wasn’t applied, so the seasonal changes of soil exchangeable calcium content were similar in all experimental plots in 2016 (Table 9). The concentration of the nutrient increased to the end of July and then dropped to the initial level. The concentration in soil of “Lobo” plots was lower than in “Zhigulevskoye” experimental site.

The concentration of exchangeable soil calcium had other dynamic changes in 2017 compared to the previous year (Table 10). The content of calcium in the soil of “Zhigulevskoye” plots had not significantly changed during the growing season. In the “Lobo” experimental sites, the soil calcium concentration increased from the end of May until the end of July, and only then it was stable until the end of the season.

### 3.4. Seasonal Changes of Leaf Potassium Status and Its Relationship with Yield

Dynamic changes of the potassium concentration in leaves of each cultivar were similar in all treatments in 2016 (Table 5) and 2017 (Table 11). The potassium content decreased during the growing season until the harvest and then recovered at the end of September. Such potassium dynamics in apple leaves are typical for the Tambov region [23]. There are some literature reports about the decrease in leaf potassium concentration [24]. The potassium leaf concentration depends on weather conditions: lack of rainfall and high temperatures can reduce the potassium leaf content [25], crop load: leaf potassium partly migrates to fruits because of the high mobility, and a strong need for ripening fruits.

In 2016, cv. “Zhigulevskoye” had a strong positive relationship between the yield and leaf potassium status, only May 30. Then, it became a weak negative correlation, and to the time of harvesting, this relationship was a close negative (Figure 3a). It confirmed our statement that in the period of fruit growth and harvesting, the potassium amount increased due to translocation from leaves.

The trend of seasonal correlation changes practically repeats the dynamics of the potassium leaf status of “Zhigulevskoye” trees during the growing season (Table 12). The negative peak of the relationship between yield and potassium leaf status of the cv. “Lobo” was in September and occurred because of later fruit ripening. Dynamic changes of potassium content in apple leaves in 2017 were, in general, similar to the previous 2016.

The character of dynamical relationship between the leaf potassium and yield had significant differences between the cultivars in 2017 (Figure 4). The variations in the “Zhigulevskoye” correlation coefficient during the growing season were similar like in the previous year. Cv. “Lobo” had a positive relationship between the potassium content and yield, not only at the start of vegetation, but also in July–August.

Because the fertilizer use result is more visible in the second and further years of the application than in the first year, we suggest that the developed fertigation program was more suitable to the requirements of cv. “Lobo”. The correlation between leaf potassium content and yield was negative in the last stage of fruit maturing in September. We can note that despite some differences, primarily associated with both cultivars’ ripening time, the leaf potassium concentration positively correlated with the yield only at the end of May. This means that the amount of soil potassium must be sufficient for plant development, first of all, at this time.

As the fruits are grown and ripened, the leaf potassium content decreased, and the more was yield, the more significant was leaf potassium reduction in growing season 2016. In 2017, seasonal changes differed between the cultivars, but both cultivars had two peaks of relationship: positive in May and negative at harvest.

### 3.5. Seasonal Changes of Leaf Calcium Status and Its Relationship with the Potassium Application Rate

Leaf calcium increased in June in treatments with relatively low potassium rates (G1), but this tendency was better expressed in G2. The intensity of these processes varied by cultivar in separate years of the study. The rise of calcium concentration in the leaves of cv. “Zhigulevskoye” was stronger in June 2016 (Table 13). It could be connected with weather conditions: in 2016, it was relatively too much precipitation in April–June and August, conversely, the rainfall was lower than usual. The lack of rain could not be a limiting factor in the irrigated orchard, but waterlogging on heavy soil have some negative impact of nutrient availability and its absorption because of lower activity of the root system. Certainly, crop load difference had some impact on the distribution of nutrients with the plant organism. Different crop load also had some influence on the distribution of absorbed nutrients within the trees. For instance, after a high yield in on-year in the next one, the calcium fruit status was significantly lower than usual [26].

Many authors report about the increase in calcium concentration during the growing season [24,27]. Considering seasonal changes of calcium content in leaves, we should take into account that calcium accumulates in vacuoles as oxalates in old leaves, and is inaccessible to various physiological processes. For analysis were sampled leaves located in the last one-third of shoots, i.e., relatively young leaves. The content of calcium in them varied in different parts of the growing season. The content of calcium in cv. “Zhigulevskoye” leaves in the Control without soil potassium application increased stable from May until the end of September. When potash fertilizers were applied, the calcium leaf status reduced and then significantly increased to the end of the growing season.

The calcium content in cv. “Lobo” leaves generally was higher than in “Zhigulevskoye” ones in May 2016. The rise of leaf calcium concentration was by both cultivars in June, but then was a slight reduction in July. In K25 and K35 treatments (G1) the calcium leaf status increased in July. Further, we marked a significant decrease in calcium leaf status in August. As a rule, the more was potassium fertigation rate, the concentration of leaf calcium decreased; the highest calcium content was in the Control treatments (without potassium soil application).

We marked a significant decrease in the potassium leaf status in G1 treatments of cv. “Zhigulevskoye” in August 2017 (Table 14). The lowest concentration was when the maximum potassium fertigation rate was applied (K45), and the highest content of leaf calcium was in the Control treatment (without potassium soil application). In G2, the content of leaf calcium also decreased, especially in K50.

The calcium leaf status of cv. “Lobo” in G1 was higher than in G2 in August–September 2017, i.e., the increase of potassium fertigation rate stimulated reduction of calcium leaf status also in cv. “Lobo” in the period of the highest requirement.

We calculated seasonal changes in the correlation coefficients between the leaf calcium content and potassium fertigation rate during the growing season (Figure 5 and Figure 6).

The experimental plots for cv. “Lobo” and cv. “Zhigulevskoye” had slightly different soil conditions; despite this, both cultivars displayed the same relationship: as the potash application rate increased, the content of calcium in leaves decreased.

On the plots of cv. “Zhigulevskoye”, the fertilizer rate was reduced already in August’s second decade (approximately 2 weeks before harvest). We did not find a relationship at the end of this month. On the sites of later cultivar “Lobo”, the fertigation rates decreased in September, and such correlation ended one month later.

To the end of June 2017, cv. “Zhigulevskoye” had a strong negative correlation; the relationship for cv. “Lobo” was weak and also negative. Then, in August, the situation changed conversely; “Lobo” had a strong negative correlation and “Zhigulevskoye” not. We think that it was because of fertigation distribution on earlier and later ripening cultivars. At the end of the season, there was no relationship with both cultivars.

### 3.6. The Content of Potassium in Apple Fruits and Its Relationship with Yield

The potassium content in cv. “Zhigulevskoye” fruits in 2016 was significantly higher than in 2017 (Table 15). The nutrient concentration changed so much because the fruit load in 2017 was considerably higher. The potassium status of “Lobo” fruits was less than “Zhigulevskoye” ones in 2016. But this situation changed completely in 2017—the yield of cv. “Zhigulevskoye” was more than cv. “Lobo” in 2017, so it could be why the “Zhigulevskoye” fruits had lower potassium fruit status than “Lobo”.

### 3.7. The Content of Calcium in Apple Frits and Its Relationship with Potassium Application Rate

The content of calcium in apple fruits of all treatments was not lower than 0.033% d.m. by both cultivars in 2016 (Table 16). But in the Control, the content of calcium was much higher—0.043% (cv. “Zhigulevskoye”) and 0,050% (cv. “Lobo”). The concentration of fruit calcium had no significant differences by cv. “Zhigulevskoye” (varied from 0.035 to 0.038) in 2016. The level of calcium in cv. “Lobo” fruits was significantly lower in G1 and G2 than in the Control. The decrease of potassium fertilizer rate stimulated higher calcium fruit status by both cultivars, but it was not significant. The use of potassium fertilizer provided a significant yield increase from the one side and decrease of soil calcium availability from the other side.

In 2017, the low calcium fruit status occurred in treatments with cv. “Zhigulevskoye” compared to 2016. This year, “Lobo” fruits had no significant difference in calcium status with the previous year. Perhaps, such a big difference between the cultivars was because of yield increase. The yield gain of cv. “Zhigulevskoye” in 2017 compared to 2016 was much higher than cv. “Lobo”.

Therefore, the differentiation of potassium fertilization rate according to crop load did not significantly impact calcium status of “Zhigulevskoye” fruits in 2016. But in 2017, the content of calcium was essential higher in G2 treatments K30 and K40 then in G1. The fruit calcium content in K50 treatment was also significantly more than in G1 treatment K35.

The reduction of potassium fertigation rates stimulated the increase in fruit calcium status of cv. “Lobo” in 2016. The next year, this reduction of 2016 provided specific fruit calcium content in G2, probably because of stored nutrient in plant tissues, despite the increase of potassium fertigation rates in 2017.

A K/Ca ratio is significant for the storability of fruits. According to Cheng [2] advice the value of this ration shouldn’t exceed 25. The K/Ca ratio in cv. “Zhigulevskoye” fruits was more than the recommended value in all G1 treatments in 2016 (Table 17). In G2 treatments with potassium fertigation rates reduced according to actual crop load, the ratio was in optimal limit and quite suitable for storage: in G2, the ratio was on the Control level. Cv. “Lobo” also had ratio values in G2 treatments lower as in G1 in this year, but the K/Ca ratio was in the recommended limit in both groups.

Potassium content in fruits was lower because of high yields, and both cultivars had a ratio value within the recommended limits in most cases in 2017. Thus, the correction of the potassium fertigation rate according to actual crop load supported a significant decrease in the K/Ca ratio. These statements are essential for good fruit storability and reducing environmental pollution with excess fertilizers.

## 4. Discussion

There are many reports in the literature about the positive impact of potash fertilizer on yield growth [28]. Nevertheless, there exists a problem in clarifying the potassium application rates. The concentration of soil potassium is not stable during the growing season. It depends on many factors: root absorption intensity, soil humidity (in dry soil, the content of available potassium is less), soil structure, temperature, microbial activity, root exudates, etc. [29]. The potassium content in ripe apple fruits is relatively high (compared to the other primary nutrients) and is similar to its concentration in apple leaves. It could be from 0.52 to 0.80% d.m. depending on cultivar [30]. This fact determines the high demand for plants in the nutrient when fruits are ripening. There are reports that the leaf potassium status has a significant correlation with yield [31].

A high potassium supply increases the sugar content in apple fruits because of this nutrient’s vital role in carbohydrate transport [32]. However, too high rates of potassium fertilizers and too large potassium fruit status can negatively influence the fruit storability [33,34]. Because of this, it is necessary to be able to correct the fertigation program during the growing season. One of the methods is to check the leaf potassium status. The optimum value must be defined for various apple phenological stages and take in to account cultivar specificity. In Central Russia, leaf sampling for nutrients supply analyses is carried out in the first half of August [15]. However, this guidance was developed in the late 1980th when the main application of potash fertilizer was broad in late autumn. According to our study results, the determination of the status of leaf potassium at the end of May makes it possible to correct a scheduled potassium fertigation rate. The optimal leaf potassium content for cv. “Zhigulevskoye” was 1.8–2.3% d.m. and for cv. “Lobo” 1.7–2.2% d.m. The cultivar specificity was not strong, and the nutrient concentration is the general physiological sign.

The content of exchangeable potassium in experimental plot soil was practically on the optimum level (180–240 mg kg^−1^) for Central Russia [15]. Nevertheless, apple trees responded better to potassium fertilization than other nutrients [35]. The change in productivity allowed us to check our idea about modifying the potassium fertigation program connected with actual crop load. One of the main principles for fertilizing is the proper demand-supply relationship [2]. Therefore, in 2016, with a lower number of fruits, we reduced, and in 2017, with a larger count of fruits, we increased the potassium fertigation rates. As leaf and fruit potassium concentration decreases at season progress [5] while the crop load increases, the demand for potassium is higher, the fertigation rates could be increased. Otherwise, lower potassium rates could be applied when crop load is smaller, we must meet the tree requirements.

Our hypothesis was partly confirmed in the cv. “Lobo” orchard already in the first year of the study: the correction of potash fertilization rates according to actual crop ensured a high yield. The reduction of potash fertigation rate in 2016 provided a yield of cv. “Zhigulevskoye” at maximum level and saved up to 20 kg ha^−1^ potassium fertilizer.

The correction of the potash fertigation rate according to the actual crop load allowed us to evaluate potassium need not only by the leaf content, but also to forecast its increasing value. One possible reason for the difference in seasonal changes of leaf potassium status in various years of the research could be the significant yield increase in 2017 (1.5 times compared to 2016). In 2016, the variations among the treatments were considerably less than in 2017. The weather conditions also varied in these years. In 2017, the beginning of the growing season was postponed by ten days because of late snowmelt and cold spring (in 12 April 2016, 21 April 2017). This was the reason for the violation of the phenological stage development. The air temperature in May and June was less than many year averages (12.3 °C < 16.3 °C, 15.9 °C < 22.0 °C, respectively). The increase in potassium fertigation rate in these conditions allowed to boost significantly the amount of available nutrient in the main root zone. The enhancement of potassium nutrition in 2017 stimulated the realization of yield potential.

The original content of exchangeable potassium in cv. “Lobo” sites was slightly lower than in cv. “Zhigulevskoye” plots because of the different soil structures. Despite the short distance between the plots (about 200 m), the soil of the cv. “Lobo” site has more clay particles, which could absorb potassium, and for some time, it would not be available for plants because such soil has the highest potassium adsorption ratio [36].

During the vegetation period, especially during fruit ripening, it is crucial to control the K/Ca ratio for good fruit storability. The optimum value of the K/Ca ratio in apple peel since small fruit (40–60 g) before harvesting should not be more than 11–13 depending on the cultivar [37]. There is also evidence in the literature that the increased potassium supply stimulated more intensive fruit coloring and increased fruit firmness in cv. Brabern/M9. This cultivar is susceptible to bitter pit, but later, during fruit storing, the development of this disorder was not noted [38].

One of the most critical indicators determining an apple fruit storability and resistance to diseases like bitter pit, sunburn, etc., is an adequate supply of calcium and optimal calcium fruit status. Gudkovskii et al. [39] concluded that the calcium content in fruit pulp for good storability, even by optimal storage conditions, should not be lower than 5 mg 100 g^−1^ (which approximately corresponds to the level of 0.030% d.m.) in the study with well-known cultivars in Central Russia.

The highest calcium fruit status was in the Control treatments of both cultivars. We think that such a result was because those trees received more soil calcium than plants in other treatments, but at the same time, these trees also got calcium from foliar fertilizer. According to the research results, many authors concluded that evaluation of potassium and calcium nutrition, in terms of further storage, must be connected to the K/Ca ratio [40,41]. The K/Ca fruit ratio should also be grounds for nutrition program development and not only the content of calcium and potassium in soil and leaves.

When fruits are maturing the main task is to provide the opportunities for developing calcium fruit status required for good storability and suppression of physiological disorder development [42,43]. To a large extent, the solution to this problem is facilitated by foliar fertilization with calcium preparations during the growing season [44]. However, according to many studies, not leaf treatments but soil calcium plays a more significant role in forming calcium reserves in plant organs [18]. Moreover, foliar calcium treatments do not always positively affect calcium fruit status, which increases the value of soil calcium absorption [45]. A plant used to make reserves the soil absorbed calcium, and mobilization of stored nutrient could be an essential source for set fruits immediately after blossoming [46].

## 5. Conclusions

According to the actual crop load, the potash fertilizer rate correction is possible only by regular application in compliance with actual soil and leaves content. The optimum value of the potassium apple leaf status in Central Russia was 1.7–2.2% at the end of May. Further research is needed to clarify the potassium rate correction algorithm during the growing season by crop load monitoring with remote methods. In our study, the amount of calcium in apple leaves decreased when yield increased only in cv. “Zhigulevskoye”. Cv. “Lobo” did not have such a clear trend. The correction of potassium fertigation rate depending on actual crop load stimulated the increase of calcium content in fruits, which led to a reduction of the K/CA ratio to an acceptable level (<20). Further research combined with apple storing must evaluate potassium rate correction’s effect on the fruit store.

## Figures and Tables

**Figure 1:**
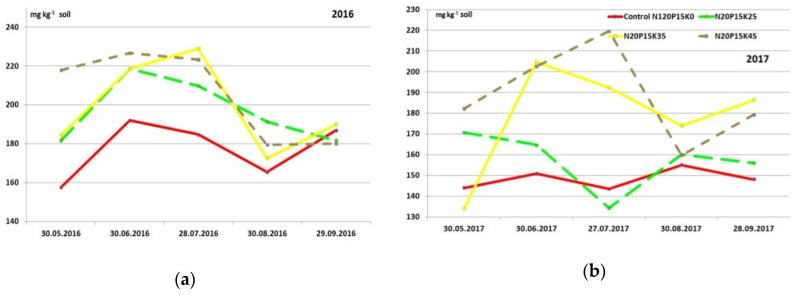
Seasonal changes of the soil exchangeable potassium content, cv. “Zhigulevskoye” plots: (**a**) 2016 and (**b**) 2017 (only G1 unchangeable K rates).

**Figure 2:**
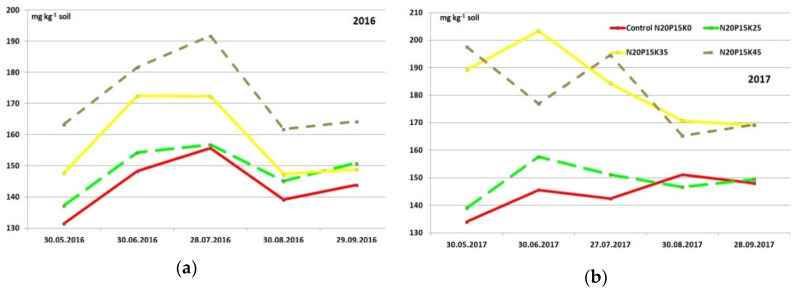
Seasonal changes of the soil exchangeable potassium content, cv. “Lobo” plots: (**a**) 2016 and (**b**) 2017 (only G1 unchangeable K rates).

**Figure 3:**
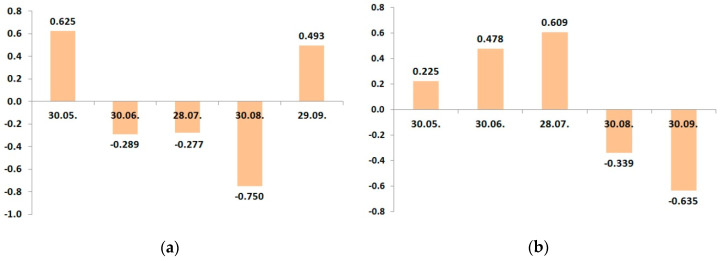
Dynamic changes of correlation coefficients between the potassium leaf status and yield in growing season 2016: (**a**) cv. “Zhigulevskoye”; (**b**) cv. “Lobo”.

**Figure 4:**
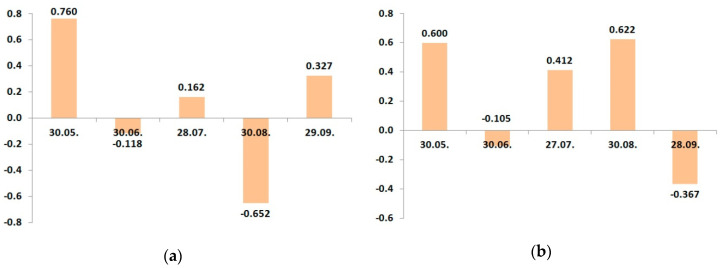
Dynamic changes of correlation coefficients between the potassium leaf status and yield in growing season 2017: (**a**) cv. “Zhigulevskoye”; (**b**) cv. “Lobo”.

**Figure 5:**
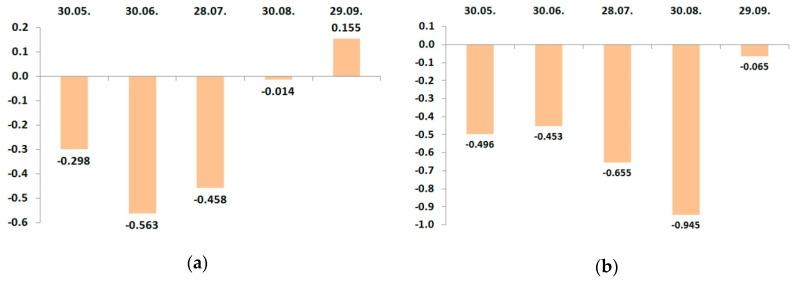
Dynamic changes of correlation coefficients between the leaf calcium status and potassium fertigation rates during the growing season of 2016: (**a**) cv. “Zhigulevskoye”; (**b**) cv. “Lobo”.

**Figure 6:**
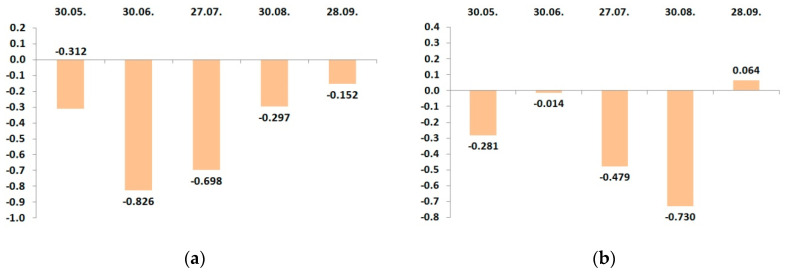
Dynamic changes of correlation coefficients between the leaf calcium status and potassium fertigation rates during the growing season of 2017. (**a**) cv. “Zhigulevskoye”; (**b**) cv. “Lobo”.

**Table 1:** Soil aggregation.

Plot	Layer, cm	Fraction Sizes, mm					
		>5	5–3	3–1	1–0.5	0.5–0.25	<0.25
		% of the Studied Soil Sample					
Cv. “Zhigulevskoye”	0–30	3.53	0.29	5.76	37.53	13.96	38.93
	30–60	0.00	0.14	7.45	35.78	15.24	41.39
Cv. “Lobo”	0–30	2.17	0.36	2.92	27.63	20.77	46.15
	30–60	0.00	0.00	2.95	33.66	19.80	43.59

**Table 2:** Experiment design.

G1, 2016, 2017	G2, 2016	G2, 2017
N_20_P_15_K_25_ N_20_P_15_K_35_ N_20_P_15_K_45_	N_20_P_15_K_20_ N_20_P_15_K_25_ N_20_P_15_K_30_	N_20_P_15_K_30_ N_20_P_15_K_40_ N_20_P_15_K_50_

**Table 3:** Foliar nutrition program.

Stages	54;55	57;61;67	71; 72;74	76;78;81
Sprayings	N, P, K, Mg, Fe, Mn, Zn, Cu, Mo, amino acid product	N, P, K, B, Mg, Fe, Mn, Zn, Cu, Mo	N, P, K, Mg, Fe, Mn, Zn, Cu, Mo	N, P, K, Mg, Fe, Mn, Zn, Cu, Mo

**Table 4:** Average monthly air temperatures (°C) during the growing season in 2016 and 2017, according to the Michurinsk Meteorological Station.

Month	Many Year Averages of Mean Air Temperature (1965–2015)	Year			
		2016		2017	
		Temperature, °C	Variation	Temperature, °C	Variation
April	6.8	9.5	2.7	7.2	0.4
May	14.5	14.8	0.3	13.3	−1.2
June	18	18.8	0.8	18.3	0.3
July	19.4	21.9	2.5	19.4	0.0
August	18.1	21.3	3.2	20.4	2.3
September	12.3	11.7	−0.6	14.0	1.7
October	5.1	5.1	0.1	5.5	0.4
Mean IV–X	13.5	14.7	1.2	14.0	0.5

**Table 5:** Monthly sums (mm) of precipitation during the growing season in 2016 and 2017, according to Michurinsk Meteorological Station.

Month	Many Year Average Sums of Precipitation (1965–2015)	Year			
		2016		2017	
		Precipitation, mm	% of Norm	Precipitation, mm	% of Norm
April	37.1	104.6	281.9	28.2	76.0
May	52.4	126.7	241.8	27.9	53.2
June	55.7	83.5	149.9	30.9	55.5
July	69.9	56.7	81.1	106.2	151.9
August	60.0	78.1	130.2	74.9	124.8
September	55.5	39.0	70.3	20.0	36.0
October	46.2	24.8	53.7	47.2	102.2
Mean IV–X	53.8	73.3	136.2	47.9	159.2

**Table 6:** Development of productivity components under the effect of various rates of potassium fertilization and fertigation approaches in 2016.

Group	Treatments	Number of Clusters	Number of Small Fruits (20–25 mm)	Number of Fruits (40–45 mm)
cv. “Zhigulevskoye”				
	Control N_20_P_15_	189	101	83
G1 (unchangeable K rates)	N_20_P_15_K_25_	177	94	85
	N_20_P_15_K_35_	170	90	81
	N_20_P_15_K_45_	181	96	86
G2 (season adjusted K rates)	N_20_P_15_K_20_	175	89	82
	N_20_P_15_K_25_	188	97	87
	N_20_P_15_K_30_	194	104	91
LSD_05_		12	6	4
cv. “Lobo”				
	Control N_20_P_15_	152	80	55
G1 (unchangeable K rates)	N_20_P_15_K_25_	146	75	53
	N_20_P_15_K_35_	140	70	51
	N_20_P_15_K_45_	144	76	58
G2 (season adjusted K rates)	N_20_P_15_K_20_	136	68	52
	N_20_P_15_K_25_	143	72	55
	N_20_P_15_K_30_	147	75	55
LSD_05_		9	4	4

**Table 7:** Development of productivity components under the effect of various rates of potassium fertilization and fertigation approaches in 2017.

Group	Treatments	Number of Clusters	Number of Small Fruits (20–25 mm)	Number of Fruits (40–45 mm)
cv. “Zhigulevskoye”				
	Control N_20_P_15_	224	126	107
G1 (unchangeable K rates)	N_20_P_15_K_25_	221	120	105
	N_20_P_15_K_35_	216	127	115
	N_20_P_15_K_45_	228	143	121
G2 (season adjusted K rates)	N_20_P_15_K_30_	224	113	104
	N_20_P_15_K_40_	218	122	110
	N_20_P_15_K_50_	233	135	125
LSD_05_		22	13	9
cv. “Lobo”				
	Control N_20_P_15_	212	108	75
G1 (unchangeable K rates)	N_20_P_15_K_25_	224	111	76
	N_20_P_15_K_35_	218	114	81
	N_20_P_15_K_45_	215	119	89
G2 (season adjusted K rates)	N_20_P_15_K_30_	212	101	77
	N_20_P_15_K_40_	216	106	81
	N_20_P_15_K_50_	202	118	91
LSD_05_		18	8	5

**Table 8:** Effect of various potassium fertilization rates and fertigation approaches on apple yield, T ha^−1^.

Group	Treatments	cv. “Zhigulevskoye”	cv. “Lobo”
2016			
	Control N_20_P_15_	26.3	19.3
G1 (unchangeable K rates)	N_20_P_15_K_25_	28.1	22.7
	N_20_P_15_K_35_	27.0	21.8
	N_20_P_15_K_45_	29.1	22.1
G2 (season adjusted K rates)	N_20_P_15_K_20_	27.8	21.4
	N_20_P_15_K_25_	28.8	22.9
	N_20_P_15_K_30_	28.6	24.6
LSD_05_		2.2	1.7
2017			
	Control N_20_P_15_	36.3	28.8
G1 (unchangeable K rates)	N_20_P_15_K_25_	38.1	35.8
	N_20_P_15_K_35_	41.9	38.9
	N_20_P_15_K_45_	48.4	39.8
G2 (season adjusted K rates)	N_20_P_15_K_30_	44.7	40.8
	N_20_P_15_K_40_	43.2	41.1
	N_20_P_15_K_50_	52.6	44.3
LSD_05_		3.0	3.2

**Table 9:** Seasonal variations of the exchangeable soil calcium content in the experimental plots of different cultivars in 2016, Mmol 100 g^−1^.

Treatments	30.05	30.06	28.07	30.08	29.09
cv. “Zhigulevskoye”					
Control N_20_P_15_	22.2	24.2	24.4	23.5	22.2
N_20_P_15_K_25_	20.5	26.4	24.4	24.3	23.0
N_20_P_15_K_35_	21.5	21.9	24.0	20.9	20.7
N_20_P_15_K_45_	21.0	24.6	29.4	22.5	23.0
LSD_05_	2.1	1.9	2.8	2.2	1.7
cv. “Lobo”					
Control N_20_P_15_	17.8	15.5	19.6	20.1	18.4
N_20_P_15_K_25_	17.9	15.7	21.6	20.6	21.3
N_20_P_15_K_35_	16.7	13.8	20.1	21.7	22.1
N_20_P_15_K_45_	17.3	16.8	18.3	18.2	23.2
LSD_05_	1.4	1.9	2.3	2.0	2.4

**Table 10:** Seasonal variations of the exchangeable soil calcium content in the experimental plots of different cultivars in 2017, Mmol 100 g^−1^.

Treatments	30.05	30.06	27.07	30.08	28.09
cv. “Zhigulevskoye”					
Control N_20_P_15_	19.6	18.8	19.1	18.2	18.8
N_20_P_15_K_25_	18.7	18.6	17.2	18.2	18.2
N_20_P_15_K_35_	19.4	18.2	17.5	18.4	17.9
N_20_P_15_K_45_	18.2	17.9	17.0	19.3	17.3
LSD_05_	1.8	1.6	2.0	1.5	1.5
cv. “Lobo”					
Control N_20_P_15_	15.7	15.6	21.4	20.9	20.1
N_20_P_15_K_25_	15.9	15.7	21.0	21.4	22.2
N_20_P_15_K_35_	16.3	20.1	21.7	22.1	19.9
N_20_P_15_K_45_	15.5	20.6	20.1	17.6	20.2
LSD_05_	1.4	1.6	1.8	1.6	2.2

**Table 11:** Seasonal changes of leaf potassium content under the influence of different potassium fertilization rates and fertigation approaches in 2016, % d.m.

	Control N_20_P_15_	N_20_P_15_K_25_	N_20_P_15_K_35_	N_20_P_15_K_45_	N_20_P_15_K_20_	N_20_P_15_K_25_	N_20_P_15_K_30_	LSD_05_
cv. “Zhigulevskoye”								
30.05.	1.81	1.89	1.74	1.97	1.76	1.92	1.73	0.10
30.06.	1.61	1.21	1.29	1.56	1.28	1.21	1.26	0.08
28.07.	1.36	1.09	1.32	1.33	1.33	1.29	1.24	0.08
30.08.	1.22	1.05	1.00	0.98	0.94	0.88	0.98	0.06
29.09.	1.30	1.27	1.31	1.52	1.12	1.37	1.44	0.08
cv. “Lobo”								
30.05.	1.67	1.77	1.78	1.95	1.71	1.73	1.68	0.11
30.06.	1.54	1.65	1.77	1.67	1.63	1.68	1.61	0.10
28.07.	1.12	1.34	1.42	1.24	1.42	1.51	1.30	0.08
30.08.	1.04	1.07	1.03	1.13	0.85	0.88	0.79	0.05
29.09.	1.15	0.91	1.09	1.03	0.85	0.87	0.96	0.06

**Table 12:** Seasonal changes of the apple leaf potassium content under the influence of different potassium fertilization rates and fertigation approaches in 2017, % d.m.

	Control N_20_P_15_	N_20_P_15_K_25_	N_20_P_15_K_35_	N_20_P_15_K_45_	N_20_P_15_K_30_	N_20_P_15_K_40_	N_20_P_15_K_50_	LSD_05_
cv. “Zhigulevskoye”								
30.05.	1.88	1.84	1.96	2.18	1.81	1.93	1.88	0.08
30.06.	1.52	1.20	1.23	1.30	1.23	1.34	1.38	0.07
27.07.	1.52	1.18	1.21	1.42	1.15	1.12	1.29	0.07
30.08.	0.86	0.90	0.88	0.79	0.83	0.79	0.76	0.06
28.09.	1.35	1.02	1.10	1.33	1.15	1.11	1.23	0.06
cv. “Lobo”								
30.05.	1.92	1.99	2.07	2.22	1.93	1.92	1.99	0.11
30.06.	1.76	1.82	1.53	1.98	1.68	1.64	1.61	0.09
27.07.	1.22	1.31	1.16	1.54	1.24	1.35	1.3	0.05
30.08.	0.76	0.97	0.89	0.88	0.99	0.94	1.06	0.06
28.09.	1.95	1.74	1.91	1.84	1.65	1.56	1.61	0.08

**Table 13:** Seasonal changes of the calcium leaf status under the influence of different potassium fertilization rates and fertigation approaches in 2016, % d.m.

	Control N_20_P_15_	N_20_P_15_K_25_	N_20_P_15_K_35_	N_20_P_15_K_45_	N_20_P_15_K_20_	N_20_P_15_K_25_	N_20_P_15_K_30_	LSD_05_
		G1 (unchangeable K rates)			G2 (season adjusted K rates)			
“Zhigulevskoye”								
30.05.	1.13	1.09	1.09	1.08	1.08	1.03	1.14	0.06
30.06.	1.50	1.50	1.46	1.42	1.56	1.55	1.43	0.08
28.07.	1.32	1.24	1.22	1.26	1.44	1.36	1.25	0.10
30.08.	1.15	1.19	1.15	1.14	1.22	1.10	1.05	0.08
29.09.	1.90	2.07	1.99	1.98	1.93	1.81	1.74	0.11
“Lobo”								
30.05.	1.35	1.27	1.26	1.27	1.27	1.17	1.24	0.07
30.06.	1.78	1.74	1.78	1.65	1.77	1.6	1.64	0.08
28.07.	1.69	1.68	1.54	1.43	1.58	1.43	1.37	0.07
30.08.	1.37	1.23	1.14	1.08	1.31	1.28	1.22	0.07
29.09.	1.67	1.56	1.63	1.67	1.62	1.55	1.59	0.08

**Table 14:** Seasonal changes of the calcium leaf status under the influence of different potassium fertilization rates and fertigation approaches in 2017, % d.m.

	Control N_20_P_15_K_0_	N_20_P_15_K_25_	N_20_P_15_K_35_	N_20_P_15_K_45_	N_20_P_15_K_30_	N_20_P_15_K_40_	N_20_P_15_K_50_	HCP_05_
		G1 (unchangeable K rates)			G2 (season adjusted K rates)			
cv. “Zhigulevskoye”								
30.05.	1.34	1.36	1.32	1.36	1.35	1.34	1.30	0.07
30.06.	1.70	1.36	1.48	1.44	1.38	1.34	1.24	0.08
27.07.	1.57	1.65	1.55	1.24	1.27	1.26	1.22	0.08
30.08.	1.28	1.20	1.18	1.24	1.19	1.17	1.26	0.08
28.09.	1.59	1.59	1.71	1.52	1.65	1.66	1.54	0.09
cv. “Lobo”								
30.05.	1.23	1.19	1.25	1.21	1.23	1.24	1.18	0.05
30.06.	1.47	1.44	1.51	1.66	1.45	1.48	1.30	0.08
27.07.	1.25	1.22	1.27	1.19	1.24	1.24	1.21	0.08
30.08.	1.15	1.02	1.07	1.00	1.06	1.09	1.03	0.07
28.09.	1.26	1.25	1.22	1.19	1.24	1.32	1.30	0.07

**Table 15:** The effect of different potassium fertilization rates and fertigation approaches on the potassium content in apple fruits, % d.m.

Group	Treatments	cv. “Zhigulevskoye”	cv. “Lobo”
2016			
	Control N_20_P_15_	0.97	0.80
G1 (unchangeable K rates)	N_20_P_15_K_25_	1.12	0.86
	N_20_P_15_K_35_	1.04	0.82
	N_20_P_15_K_45_	0.96	0.92
G2 (season adjusted K rates)	N_20_P_15_K_20_	0.84	0.73
	N_20_P_15_K_25_	0.87	0.73
	N_20_P_15_K_30_	0.83	0.75
LSD_05_		0.07	0.06
2017			
	Control N_20_P_15_	0.58	0.73
G1 (unchangeable K rates)	N_20_P_15_K_25_	0.68	0.71
	N_20_P_15_K_35_	0.64	0.69
	N_20_P_15_K_45_	0.61	0.75
G2 (season adjusted K rates)	N_20_P_15_K_30_	0.55	0.68
	N_20_P_15_K_40_	0.57	0.71
	N_20_P_15_K_50_	0.62	0.78
LSD_05_		0.04	0.05

**Table 16:** The effect of different potassium fertilization rates and fertigation approaches on the calcium fruit status, % d.m.

Group	Treatments	cv. “Zhigulevskoye”	cv. “Lobo”
2016			
	Control N_20_P_15_	0.043	0.050
G1 (unchangeable K rates)	N_20_P_15_K_25_	0.035	0.034
	N_20_P_15_K_35_	0.037	0.031
	N_20_P_15_K_45_	0.038	0.033
G2 (season adjusted K rates)	N_20_P_15_K_20_	0.039	0.041
	N_20_P_15_K_25_	0.038	0.034
	N_20_P_15_K_30_	0.036	0.037
LSD_05_		0.003	0.004
2017			
	Control N_20_P_15_	0.037	0.040
G1 (unchangeable K rates)	N_20_P_15_K_25_	0.024	0.033
	N_20_P_15_K_35_	0.021	0.035
	N_20_P_15_K_45_	0.024	0.029
G2 (season adjusted K rates)	N_20_P_15_K_30_	0.028	0.034
	N_20_P_15_K_40_	0.028	0.031
	N_20_P_15_K_50_	0.027	0.032
LSD_05_		0.004	0.004

**Table 17:** The effect of different potassium fertilization rates and fertigation approaches on the K/Ca ratio in apple fruits.

Group	Treatments	cv. “Zhigulevskoye”	cv. “Lobo”
2016			
	Control N_20_P_15_	22.6	16.0
G1 (unchangeable K rates)	N_20_P_15_K_25_	31.1	25.3
	N_20_P_15_K_35_	28.1	26.5
	N_20_P_15_K_45_	29.1	27.9
G2 (season adjusted K rates)	N_20_P_15_K_20_	23.3	17.8
	N_20_P_15_K_25_	22.9	21.5
	N_20_P_15_K_30_	23.7	20.3
LSD_05_		1.7	1.3
2017			
	Control N_20_P_15_	14.9	18.3
G1 (unchangeable K rates)	N_20_P_15_K_25_	28.3	21.5
	N_20_P_15_K_35_	30.5	27.6
	N_20_P_15_K_45_	25.4	25.9
G2 (season adjusted K rates)	N_20_P_15_K_30_	16.7	17.9
	N_20_P_15_K_40_	15.0	17.3
	N_20_P_15_K_50_	16.3	22.8
LSD_05_		1.5	1.3
